# Dismissing the Moral Sceptic: A Wittgensteinian Approach

**DOI:** 10.1007/s11406-016-9805-9

**Published:** 2017-01-13

**Authors:** Sasha Lawson-Frost

**Affiliations:** Philosophy Department, University College London, 19 Gordon Square, London, WC1H 0AW UK

**Keywords:** Wittgenstein, Moral scepticism, Aspect-perception, Metaethics, Philosophical investigations

## Abstract

Cartesian scepticism poses the question of how we can justify our belief that other humans experience consciousness in the same way that we do. Wittgenstein’s response to this scepticism is one that does not seek to resolve the problem by providing a sound argument against the Cartesian sceptic. Rather, he provides a method of philosophical inquiry which enables us to move past this and continue our inquiry without the possibility of solipsism arising as a philosophical problem in the first place. In this paper, I propose that Wittgenstein’s method of dismissing the Cartesian sceptic can also be applied to the problems posed by the ‘moral sceptic’, who denies the truth of all ethical or moral claims. I will argue that in the same way Wittgenstein’s focus on public language enables us to dismiss the traditional problem of other minds, a focus on public moral practices or language-games also enables us to dismiss the idea that moral claims are always ‘meaningless’, ‘false’ or ‘nonsensical’. On this account, the moral sceptic is misguided in much the same way as the solipsist who implicitly admits the existence of other minds in her practices. The moral sceptic who still engages in moral activities also implicitly admits the existence of meaningful moral positions. Wittgenstein’s dismissal of the Cartesian sceptic, as I understand it, can be broadly divided into two parts. The first part is an account of language acquisition. This part outlines how we might come to see other humans as conscious, thinking, feeling beings from a causal perspective. This suggests that we can arrive at an understanding of other minds as a primary perception itself - without needing to posit this perception as a kind of deductive or inductive hypothesis. Secondly, we can see how this relates to an epistemic model of language. This focuses on the role of language as something which consists of rule-governed activities, where the existence of other minds is embedded in our understanding of the world as a kind of grammatical rule, rather than an observational hypothesis. From both these arguments the Cartesian sceptic is, (on Wittgenstein’s account), irrelevant to some forms of philosophical inquiry. This is because the sceptic takes the existence of other minds to be a rational hypothesis/inference when it is not. I suggest that this approach can be applied to moral scepticism if we take certain normative claims as grammatical dispositions (practical and tautological), rather than rational or metaphysical propositions. Hence, the moral sceptic who offers a rational or logical critique of these moral foundations is not necessarily saying anything relevant to our practices – the moral stances which they refute as rationally meaningless were never based on purely rational or logical hypotheses in the first place.

## Introduction

Wittgenstein’s dismissal of the Cartesian sceptic is one that does not seek to resolve the problem of solipsism or Cartesian doubt. Rather, it enables us to move past this and continue our inquiry without this scepticism arising as a philosophical problem in the first place. In this essay, I propose that Wittgenstein’s method of dismissing the Cartesian sceptic can also be applied to the problems posed by the moral sceptic. In doing so, I argue that in the same way Wittgenstein’s focus on public language enables us to dismiss the traditional problem of other minds, a focus on public moral practices or language-games also enables us to dismiss the idea that moral claims are always ‘meaningless’, ‘false’ or ‘nonsensical’. This leads us to an interesting distinction. Whilst virtually no-one genuinely identifies as a solipsist, a large number of people do maintain the position that moral language is meaningless, or that there are no moral facts. I argue in this paper that the moral sceptic here is in fact misguided in much the same way as the solipsist who implicitly admits the existence of other minds in her practices. The moral sceptic who still engages in normative activities also implicitly admits the existence of meaningful moral positions. This distinction is therefore not one of philosophical content, and the focus of moral philosophy can be shifted away from the moral sceptic; much like the Cartesian sceptic.

I shall now clarify what I mean by ‘moral scepticism’. My definition of this position can be broadly summarised as the view that all moral claims are in some sense ‘meaningless’ or ‘false’, and hence ought not to be given any serious consideration in philosophical discussion. For examples of this in academic literature, we can look to error-theorists like Mackie and Royce (stating that all moral claims are false), as well as emotivists like Ayer (stating that all moral claims can be reduced to non-normative facts about our emotional/psychological dispositions) (Ayer 1936). Mackie’s account claims that “there do not exist entities or relations of a certain kind, [or any] objective values or requirements” (Mackie 1977, p. 18). Hence if someone were to make a moral claim appealing to such entities, (as most absolute moral claims seem to), this would inevitably be false. Royce develops this account in terms of the objective *normativity* required by absolute moral claims. On his account, normative reasons are “agent-relative”, and hence fail to provide the “authority” that moral frameworks necessarily command – they are hence just mistaken frameworks (Royce 2001, pp. 33–34). In this essay, I suggest that these accounts are mistaken in taking moral claims to be referring to absolute, ontological states of affairs in the first place. Just as the Cartesian solipsist mistakenly takes the existence of other minds as a rationalist claim, moral sceptics like Ayer, Mackie and Royce mistakenly take moral claims to be rational or ontological propositions. I argue that the morality that they criticise is just not the morality which most people accept (implicitly or otherwise) in our public language-games.^1^

## The Cartesian Sceptic

Wittgenstein’s dismissal of the Cartesian sceptic, as I understand it, can be broadly divided into two parts. The first part is an account of language acquisition. This part outlines how we might come to see other humans as conscious, thinking, feeling beings from a causal perspective. This suggests that we can arrive at an understanding of other minds as a primary perception itself - without needing to posit this perception as a kind of deductive or inductive hypothesis. Secondly, we can see how this relates to an epistemic model of language. This focuses on the role of language as something which consists of rule-governed activities, where the existence of other minds is embedded in our understanding of the world as a kind of grammatical rule, rather than an observational hypothesis. From both these arguments the Cartesian sceptic is, (on Wittgenstein’s account), irrelevant to some forms of philosophical inquiry. This is because the sceptic takes the existence of other minds to be a rational hypothesis/inference when it is not.

These arguments taken separately however, do not seem to provide a full account of why we ought to dismiss the Cartesian sceptic. The first part of Wittgenstein’s argument here seems very contentious if taken as a purely empirical hypothesis. Hence, if we do take the argument as such, and it turns out that Wittgenstein’s account of language acquisition doesn’t correspond to the way children do learn language, then Wittgenstein would be wrong to dismiss the Cartesian sceptic. Indeed, if we do take this argument on its own, it may well appear that Wittgenstein significantly lacks any empirical evidence to assert his observations! The second part similarly does not fully support the dismissal of the Cartesian sceptic. This is because, whilst it may offer an alternative epistemic model to Descartes’, this model alone does not offer any clear reason that we ought to accept this model and reject that of Descartes. It does not provide an account of why a Cartesian approach to philosophy would be in any way incoherent or useless – it just suggests that such an approach does not aptly correspond to the way we see the world. This then demands the question of why this should constitute a criticism such that we ought to dismiss Cartesian epistemology.

However, I would suggest that we can interpret these two parts of Wittgenstein’s argument more charitably if we focus on the how they relate to each other, rather than assessing them separately. In particular, I suggest that we can take Wittgenstein’s account of language acquisition, not as an empirical claim, but instead as a kind of analogy which leads up to his epistemic account.^2^ In this way, Wittgenstein is also able to justify the importance of his own epistemic model by providing an argument against the *coherence* of the Cartesian approach – a completely ‘private language’. My interpretation here focuses on the role of first/second/third personal thought in these arguments.

### Language Acquisition

Wittgenstein offers an account of language acquisition which contrasts with that of Augustine. Augustine’s child is pre-linguistically capable of recognising how the elders of his community react to pain-behaviours such as crying, and respond to this by manipulating their behaviour to achieve his desires (Augustine 2002; Mulhall 2007). Wittgenstein, however, suggests that this is impossible, and argues that Augustine’s child *cannot* have not have this complex mental life whereby it can recognise and respond to its own desires (Mulhall 2007). Instead, Wittgenstein suggests that the link between pain and pain behaviour is created by the elders of the community, rather than being inferred by the child himself. This is caused by a natural association on the part of the elders between pain and pain-behaviour. The child is only later able to recognise this connection when it begins to learn language and enter the community of elders where this association is already established. The pre-linguistic child, therefore, does not undergo a process of recognising his own emotional states and inferring a link between this and his behaviour. He is instead treated as part of a language-speaking community, and only in entering this community is able to identify pain behaviour as related to actions, perceptions and feelings. These links are recognised according to that community’s wider practices and language-games.

In what sense, then, is Wittgenstein’s picture more coherent than Augustine’s? I said previously that, on Wittgenstein’s account, Augustine’s child cannot possess the mental and linguistic powers required by his account of language acquisition. This can be interpreted in a number of ways: for example, it might seem like a hypothesis about the philosophy of mind or theoretical linguistics. Here, however, I am going to focus on this as an epistemological claim, whereby the understanding of Augustine’s child requires an understanding of public language which he has not yet been exposed to, and therefore can have no practical or cognitive meaning for that child. It is therefore not just practically impossible for the child to acquire language like this, according to a particular empirical hypothesis, but theoretically impossible according to Wittgenstein’s conception of meaning.

This perhaps seems clearer if we focus on the role of perspectival reasoning in Wittgenstein’s example. The pre-linguistic child is the subject of pain and various feelings from an immediate perspective (i.e. he feels pain). In order to go beyond this, however and recognise *that* he feels pain, further understanding is needed. That is, he needs to he needs to be able to see things from an outer perspective where his sensation of pain is not the only thing that exists.^3^ This perspective can be achieved, for example, by recognising that other people in his community are not in pain and that at other points in time he is not in pain.^4^ In this way, by seeing things from the perspective of a community or a member of a community, the child is able to distance himself from his own sensations and recognise them as temporal. This exposure to public language allows the child to recognise his sensations as only fulfilling a small part in the community of his peers, and not the whole of it (as it was for the pre-linguistic child without this means of distancing) (Mulhall 2007; Wittgenstein 1953).

The existence of other minds is therefore not something which we infer according to our own inner reasoning processes, but actually *precedes* them. That is, the recognition of other persons’ sensations is developed alongside a recognition of our own. The Cartesian sceptic is therefore mistaken in assuming that one’s ‘inner’ sensations are somehow epistemically prior to other minds. Without this assumption we have no reason to doubt the latter according to the former, in the way Descartes wants. (For example, it would be just as reasonable to start with other minds as the beginning of inquiry rather than our own).

### Wittgenstein’s Grammar

Wittgenstein’s dismissal of the Cartesian sceptic is reinforced by his broader account of language as a rule-governed activity. With this account, we can distinguish between two aspects of language. Firstly, there are ‘grammatical rules’ which determine and govern how we use words in specific ‘language-games’ (Wittgenstein 1953). Secondly are statements that follow from these rules and are made within the context of a language-game. The latter can be assessed (at least in part) according to the conditions outlined by the former. We can assess propositions as true/false, or right/wrong, according to the rules or practices that make them so (Arrington and Glock 1992). According to Wittgenstein’s model of how we come to recognise other minds, it appears that an understanding of other minds is not, epistemically speaking, an inference we make about the world, but is rather a grammatical presupposition that underpins all of our rational and linguistic inferences about the world.

To illustrate this, we can look to Wittgenstein’s discussion of the phrase “my sensations are private” (Wittgenstein 1953, §248). Wittgenstein draws on the intuition that we can’t understand or conceptualise what it would mean for this statement to be false. This is not to be understood just in the sense of it being impossible for us to it imagine the sentence as false, but also because the notion of sensations not being private is totally inconceivable according to the way we use these terms. We might, for instance, want to ask ‘well if sensations are not private, *what is*?’ – it seems like such a basic formulation about our sensations that in order to doubt their privacy we would have to bring into question our very conception of ‘sensations’ or ‘privacy’. The statement, according to Wittgenstein is therefore not an empirical proposition, but a *grammatical* one (Wittgenstein 1953, §251). It cannot be the former, since in order for it to be an empirical proposition, we would have to be able to illustrate, according to some independent check, what makes it empirically true or false. This cannot be done since nothing could make it empirically false – this is already determined by our use of the words themselves. Hence, there is no further criterion that I can appeal to and say ‘this is what I mean by stating that is true, rather than false’ (Arrington and Glock 1992). Instead, the statement serves to remind us how we use the concept of ‘privacy’ in the first place, in the same way that Wittgenstein’s ruler serves as a rule or guide for determining how long we take a metre to be. Grammatical rules or statements therefore serve as guides about how we use language (Wittgenstein 1953, §254).

With this in mind, we can see the existence of other minds not as an empirical proposition which can be assessed as true or false according to some further criterion, but instead as a rule governing our practices. Unlike the previous example, it is (probably) theoretically possible to doubt that ‘other people have minds’. However to do so is already to falsely assume that the existence of other minds is the kind of proposition which depends on some further criterion (e.g. rational or scientific analysis). According to Wittgenstein, positing the existence of other minds is actually a kind of rule-governing criterion itself, rather than something which is subject to some further criterion.

The fact that we seemingly can doubt the existence of other minds perhaps indicates a significant disanalogy between the other-minds example and the ‘sensations are private’ example. That is, I can plausibly imagine a scenario where all other humans are philosophical-zombies. We probably wouldn’t say that this is a logically incoherent suggestion. On the other hand, statements like ‘sensations are private’, or ‘the metre-rule is one metre long’ it seems like we simply can’t conceptualise what it would mean for these to be false, (at least without misunderstanding one of these terms). I don’t think that this distinction is very fundamental to Wittgenstein’s notion of grammar, but for the purposes of this essay the following distinction will be helpful:

#### Tautological Grammar

Grammatical rules which are true in virtue of some given definitions of terms within a language-game. Examples: ‘1’ is a number, a metre is 100 cm.

#### Practical Grammar

Grammatical rules which are not true by definition, but are always taken to be true within a language game. Examples: other people have minds, the knight is horse-shaped.

It is important to note here that this distinction only works as a pragmatic device is cases where the rules of a language-game are (artificially) set out for the purposes of philosophical inquiry. (For instance, if I were to ‘set up’ an analytical paper where the necessary definitions are clearly outlined beforehand).^5^ It seems to me that doing any more than this is impossible if we take Wittgenstein’s emphasis on the *practice* of language seriously – since on this account there are no inflexible, essential ‘definitions’ or ‘meanings’ of terms that we could appeal to in applying this distinction. To posit any definitions like this and claim that they do correspond with the realities of language would be to deny the open-texture of language. For example, take the statement ‘the knight moves in an L-shape’. This might be taken as an instance of tautological grammar if we take this to be a literal definition of ‘knight’ in chess, or it might be taken as an instance of practical grammar.

This (very qualified) distinction allows us arbitrate between grammatical rules which serve as reminders of what we ‘mean’ by terms, and act like definitions, (tautological grammar), and those which we implicitly take to be true, for instance through aspect-perception, (practical grammar). The latter could plausibly be questioned if these grammatical remarks are taken to be propositional theses, even if they are not actually used as such in our practices. For instance, even if I take it as given in all practical scenarios that other people are not philosophical zombies, and see ‘that’ others exist rather than inferring it, it is not totally incoherent to entertain the notion that this perception does not reflect the way things are. It is simply not pertinent to most practical inquiry. The significance of this can be seen if we look to the way grammatical assumptions evolve with our language practices. Whilst practical grammar might plausibly be questioned, rejected and updated according to our practices. It may be that we need to modify the assumptions we make about other minds in certain activities contextually. For instance, we might want to overcome biases that make us assume people think or experience in the same way (e.g. typical mind fallacy). New evidence might arise in particular language games which means these initial presuppositions about other minds are problematic or unhelpful. For instance, we may need to question our initial views about the nature of other minds when studying sense perception, or the way people reason. The same cannot be done for tautological grammar since it just isn’t possible to conceive of any way these grammatical remarks could be false.

This process of modification, however, only needs to be done in accordance with the aims of the language-game in question, rather than according to some absolute degree of consistency. Our everyday practices depend on making the assumption that other minds do exist and that solipsism is wrong. This means that the sceptic who rejects the existence of other minds but still engages in these social practices is, in a sense, still committed to the existence of other minds.

Hence, whilst the Cartesian sceptic might say that the existence of other minds not grounded in pure reason, this really doesn’t matter as far as our practices are concerned. The kind of assumption we make about other minds is not, as the rationalist supposes, a proposition that can be proven to be true or false. The sceptic is therefore not actually attacking the beliefs that most people implicitly hold and that are ingrained in our practices. Therefore, if we are engaging in the kind of philosophy which seeks to analyse the structure and upshots of our existing language games, we can push the concerns of the solipsist aside. Whilst practical grammar might plausibly be questioned, rejected and updated according to our practices.

## The Moral Sceptic

We have so far outlined a way of dismissing Cartesian scepticism for the purpose of progressing (a particular kind of) philosophical inquiry, without refuting it on its own rationalist grounds. I now suggest that this approach can be applied to moral scepticism if we take certain normative claims as grammatical dispositions (practical and tautological), rather than rational or metaphysical propositions. Hence, the moral sceptic who offers a rational or logical critique of these moral foundations is not necessarily saying anything relevant to our practices – the moral stances which they refute as rationally meaningless were never based on purely rational or logical hypotheses in the first place.

There are clear grounds for suggesting that at least some moral claims will be of the grammatical form I have described. This is shown simply by the fact that we *do* engage in moral practices and language-games: we criticise people for doing ‘the wrong thing’ and say that they ‘ought’ to do ‘the right thing’. We sometimes experience or express a sense of moral repulsion or abhorrence at events we think of as wrong. These practices therefore have to be based on some kind of conceptual (grammatical) assumptions in order to even begin inquiry where inferences about morality can be made. Without some kind of grammatical bedrock – where I simply say “this is what I do” (Wittgenstein 1953, §217) – I have no way of making the aforementioned claims at all, since there would be no framework with which I could arbitrate between these propositions and other opposing or contradictory propositions (or saying nothing at all!). We cannot take all these moral statements to be non-grammatical inferences since this would lead to an infinite regress of various justifying frameworks (i.e. this statement is the case because of x framework, and the rules/application of x framework are inferred because of y framework, which is inferred according to z framework… etc.). Since we reason in a finite amount of time, this is not humanly possible, and so we have to take some framework as grammatical, rather than inferential.

What, then, constitutes the grammar of our moral language-games? We might first look to examples of moral responses which are unjustified or phenomenological, rather than ones that are used in a way that is propositional or descriptive. An example of this can be seen with Gaita’s discussions of rhetorical moral statements. Consider the following passage:

“[A French woman] had witnessed, over a long period, a young Nazi officer sending trainloads of (mainly) children to the death camp. She said that every day since she had asked herself how it were possible for him to do it. Hers is not a question which invites an answer. It expresses a mystery at that kind of contact with evil, and that sense of mystery is connected with a sense of the reality of evil as something *sui generis*.” (Gaita 1991, p. 5)

Here, Gaita seems to be describing a kind of moral repulsion and confusion at the woman’s experience of this ‘evil’ which cannot be understood according to some further criterion or framework to define what makes it evil. Instead this evil is (somehow) recognised as such in its own right, and not (for instance) by identifying something evil about the behaviour of the Nazi officer and then inferring that he did the wrong thing. We might then suggest that the woman’s expression of mystery is one that communicates a failure to understand something so far beyond our moral practices – to the extent that she feels as though there is something that she cannot comprehend about how the Nazi officer could do these things. This lack of comprehension, then, might be understood in a very literal sense – she is faced with something that transgresses all moral boundaries determining what people can (permissibly) do. She is then perhaps in a similar position to Wittgenstein’s pre-linguistic child who cannot recognise his own sensations because he has not yet engaged in public practices. The woman is faced with a scenario which, according to her society’s existing moral codes, ought never to have happened, and she does not have the mental apparatus with which to comprehend this, (i.e. none of her previously encountered language-games can account for such events). In this sense, we might interpret some statements like *‘you can’t do that!*’, (and the corresponding confusion about ‘*how could you do that*?’), as reminders of the boundaries of our various moral spheres – to transcend this would be to step beyond or outside the boundaries of the moral language games that we play. To respond to this scenario with scepticism about the evilness of the Nazi officer’s actions, or dismiss the woman’s confusion as irrational, would therefore just miss the point – because these statements and reactions are existing parts of our core moral frameworks, not something which is up for critical discussion.

The grammar indicated here, I suggest, can be understood as a form of practical, not tautological grammar. Statements like ‘the Nazi party’s actions were wrong’ serve as reminders of how we talk about morality, and prompt a reaction in us which seems to constitute a kind of moral aspect-perception. However, this doesn’t mean that the statement couldn’t be conceivably construed as false. We can, for instance, engage in hypotheticals about what makes the statement true. Even if we are not prompted in our practices to ask *whether* the statement is true, we can ask what further grammatical statements (i.e. tautological ones) *make* it true. For instance, we might say that the Nazi officer’s actions are wrong because they involved wrongfully killing innocent children – not simply in virtue of him being a Nazi officer.

Another possible way of identifying our ‘moral grammar’ can be seen if we look to Pleasants’ discussions about moral certainty and the seriousness of killing. Pleasants argues that “the wrongness of killing must be considered a basic moral certainty because its wrongness cannot sensefully be asserted, explained or doubted and instead “serves as a fundamental condition of human morality” (Pleasants 2015, p. 202). Hence, the moral sceptic is wrong to take statements like ‘killing is wrong’ as senseful or propositional, since these are just basic moral rules which govern our moral language-games. We might then say that the moral sceptic could just be conflating a concept of ‘nonsense’ with senseless grammatical propositions. To treat these two in the same way would be like responding to the statement ‘sensations are private’ with the exclamation ‘nonsense!’ rather than (more intuitively) ‘that’s obvious’ (Wittgenstein 1953, §252). These two concepts might be qualitatively similar – neither have ‘sense’ in the sense of a relevant criterion of correctness - but for the latter, the lack of sense is not a reason to reject these claims.

I suggest, however, that Pleasants’ account also only offers an instance of practical grammar. This is shown just from the fact that in many cases we do take killing to be morally justified. This can be seen just from the way in which Pleasants qualifies this claim, referring to the claim “that it is very wrong to kill an innocent and non-threatening person, absent special excusing or justifying circumstances” (Pleasants 2015, p. 200). The implication here is that, given certain justifying circumstances, or given a ‘threatening’, ‘guilty’ victim, it would not be obvious or certain that the killing were wrong. My concern here is that, if we allow there to be extenuating circumstances where the statement ‘killing is wrong’ is false, then it seems we also have to admit some further kind of criterion which allows us to arbitrate between instances where the statement is true and when it is not. We can clearly can imagine circumstances where the statement ‘killing is wrong’ is not true, or at least not certain, and hence the claim is theoretically dubitable. In this sense, therefore, Pleasants’ does not identify an instance of basic tautological grammar. The claim ‘killing is wrong’ may be an instance of practical grammar, in that we never practically take the statement to be false, and we see *that* killing someone is wrong rather than inferring it. However, this doesn’t provide us with the kind of tautological framework that a full account of our moral grammar needs – it does not offer a definitional account of how we use terms like ‘killing’ and ‘wrong’ for example. Without this, it seems that statements like this (practical moral grammar) can still be put under scrutiny.

To develop this account further, so that we can get to grips with our tautological moral grammar, I suggest look to the ways in which the kinds of moral claims discussed by Gaita and Pleasants are adapted and modified. In this way, we can assess how a more foundational conception of how we use moral terms, (e.g. killing, should, wrong), determines our practical moral grammar. We are here looking for a particular kind of tautological grammar that could act as reminders of why certain moral claims or rules are accepted (as practical grammar) over others. By examining the way that our moral language games adapted and modified, we can perhaps account for the structure of the *normative* force behind these changes, making our moral language games distinctly ‘moral’ from the standpoint of the people practicing them.

Let’s return to Gaita’s passage. Whilst we might have a huge sense of ‘mystery’ when faced with events that our existing conceptions of morality somehow cannot account for, this does not mean our moral frameworks/languages cannot adapt or develop in response to these mysteries. For instance, since the end of WWII the name ‘genocide’ has been introduced for condemning acts like those of the Nazis, including a whole load of legal and moral implications of the use of this term. Arguably, these kinds of developments in language allow us to account for these types of evil within our existing language games, so that we can come to terms with the existence these kinds of events in the way that the woman in Gaita’s scenario was unable to. This is, of course, not to say that this sense of mystery has been completely done away with – I imagine many people do want to ask ‘how could this have happened?’ when hearing about the Holocaust – but to some extent we are able to discuss and account for these events historically and morally without being completely alienated by this sense of mystery. I am able to distance myself from this sense of mystery and engage in public moral inquiry about genocide. (In much the same way, I might be sometimes perplexed by the existence of other persons with different psychologies, but I can still talk with/about them according to public language practices).

What is significant is that these changes in moral language do not occur arbitrarily. In order to adapt our moral frameworks in this way, I suggest, we have to abstract from these initial intuitions and decide what it is that makes these intuitions the case. These claims would not, therefore, be certain in the sense of something that is not open to critical reflection. This further reflection can be examined if we investigate what is that makes us have these moralised responses to these events in the first place. Clearly some aspects of Gaita’s scenario are morally relevant, whilst some are not (e.g. it doesn’t seem to matter what time of day the Nazi officer sent the children to the camps). This leaves open the question of how we are to arbitrate or distinguish between these factors, which requires a separate framework of assessment. For instance, is this example so abhorrent and incomprehensible to the woman because the people he is sending to die are children? Because they are ‘innocent’? Because they are people? (Would it be completely different if they were adults, guilty of a crime, or animals?). With Pleasants’ scenario, we might similarly ask what it is that makes killing *killing*, (such that it is morally wrong). For instance, what makes some societies see the lynching of black people as morally permissible whilst simultaneously viewing murder as wrong? What is morally serious in both these scenarios is therefore not revealed by these grammatical remarks alone – they are not just morally significant in their own right, but against a backdrop of further moral norms.

I would suggest that the way in which these moral languages change can be understood through an underpinning structure of instrumental reasoning about things that we care about. Hence, when external events cause us to question what we consider valuable or what matters to us, we can re-evaluate our moral frameworks accordingly. This allows us to also have a further framework against which we can describe certain changes in our moral frameworks and perceptions as progressive or regressive, and beneficial or detrimental. Take, for instance, the basic intuition that killing people is wrong. I maintain that, in addition to being intuitive and (to some extent) grammatical, this is also used on a further level as a *heuristic* for the sake of valuing the lives of those we care about. On its own, the statement ‘killing is wrong’ would be totally arbitrary, and have no normative force except for the fact that it tends to be accepted by most communities (as a result of biological factors, for example). With the additional premise ‘we care about the lives of people’, our grammatical moral rules immediately do become normatively significant just from the fact that certain grammatical moral rules will fulfil this end to a greater extent than others. We can therefore make normative, as well as descriptive claims, about our moral practices, according to instrumental reasoning.^6^ For instance:

1. We care about the lives of people in our community
2. Killing people is harmful to the lives of people in our community

C: Therefore, the moral rule ‘killing is wrong’ is a good/useful rule for our community to have.

P2 here is only an intermediary rule between our values and our practice, so what is actually fundamental about our moral practices, and perhaps ‘certain’ in a Wittgensteinian sense, is our cares and values, which motivate certain grammatical rules to be used in the first place – such as those in P1. We can think of this instrumental ends or values as elements which fix the rules of language-games in a non-arbitrary fashion; this means that the rules of the game themselves are normative from the players’ perspectives, rather than just leading to normative statements (e.g. the players value the rules of the game themselves as well as being able to use these rules to say what you can or should do in these games).

We can see that without these more fundamental rules about our cares and motivations, language-game practices have no (instrumentally rational) way of being asserted in groups, since any particular set of rules can be overturned and changed completely without any sacrifice to the players. We might think of children playing a particular ball game or something where they change the rules of the game randomly every so often – if they are just trying to have fun and there is no reason why one of these sets of rules should be better than any others and no one minds the game constantly changing, there is no reason for it to become fixed in one particular way. With other games like Chess though, the rules are specifically adapted for the purposes of a certain kind of entertainment or education. People might therefore get annoyed if you keep insisting on changing the rules in the middle of a game, since this would interfere with a set-up which is already orientated towards these initial purposes. This suggests that, if people/communities are to some extent instrumentally rational and react their own cares and motivations, these should and will have a causal role in the fixation of rules in language-games.

This model allows us to explain what happens when our moral grammar changes according to this initial framework of cares and values. Say that the community in the above example only values the lives of non-black people. According to this value, the community might only understand the statement ‘killing is wrong’ in the context of non-black people, for instance if black people were not really considered ‘people’ on some level. However, if societal values change and black people become humanised within this community, then we might say that their understanding of what counts as ‘killing’ ought to be recalibrated according to the communities own instrumental ends.^7^ Whether this actually happens is more of a question of instrumental psychology, but at least on a normative level we can here say that they ought to, and have some reason to be motivated in this direction. The fact that most communities don’t have total agreement about these values perhaps also explains the disagreement faced when these values are scrutinised – for instance, if we look at what is at stake in debates about abortion, a significant amount of disagreement arguably stems from the question of what constitutes ‘life’ such that it ought not to be killed.

I therefore suggest that the practical grammar of our moral practices is up for critical discussion, rather than being irrefutable or certain. Instead, these can be criticised and modified according to the cares and values of those engaging in these practices. Now, to return to the question of the moral sceptic. According to the framework I have outlined, neither the basic moral norms of a society or the values that we assess these against are beyond the scope of sceptical inquiry in the same way that our understanding of other minds is. This is because claims such as ‘I value x’ and ‘y is wrong’ are both propositional, and hence depend on what it is we do value,^8^ and what makes it the case that y is wrong. They are therefore open to sceptical critique since their truth-value is something which depends on external factors. What perhaps is beyond scepticism, however, is the structure of instrumental reasoning which underpins this whole process of how a community governs its moral practices. Take the following statements, for instance:

- ‘I ought to act according to my own interests’
- ‘I ought to act according to my community’s interests’
- ‘I ought to follow the rules of a game if I want to play it well’

These, I suggest, are instances of tautological grammar, in that they can serve to remind/instruct us how we can talk about what we ought to do in the context of certain actions, communities or language-games. These sorts of remarks are not inferential claims about the content of morality, but can instead function as linguistic rules/reminders that are ingrained in the structural, normative nature of our moral practices and norms. This idea shares some similarities with Searle’s account of “institutional facts” in his 1964 paper ‘How to Derive “Ought” from “Is”’. An important difference between my account and Searle’s, however, is that I am dealing with the epistemology of *normative* claims, rather than *evaluative* ones^9^ As instructive ‘speech acts’ the examples I have given do not necessarily bridge the gap between ‘descriptive’ and ‘normative’ claims, (as Searle claims they do for ‘descriptive’ and ‘evaluative’ claims). Both these examples of grammar, and the claims we want to derive from them, are normative claims,^10^ (e.g. they could be spoken as commands, instructions or grammatical reminders). That is, they attempt to describe how people *ought* to be or act, rather than how they *do* act or how they *are.* To put it in Searle’s terminology, they are performative acts rather than merely descriptive ones (Searle 1964, p. 51).

If we take the above examples to be instances of tautological grammar, this does not resolve the naturalistic fallacy about normative claims.^11^ In the sense which I am concerned with, these claims are already normative, and so this does not explain how we can get normative statements out of merely descriptive ones. What I think this does show is that we do not need to resolve the naturalistic fallacy in order to progress moral inquiry and engage with moral/normative language-games. These claims can be taken for granted in most human language games,^12^ and so it does not matter if these rules cannot be deduced from some set of rational or empirical premises, (from the perspective of those in the language-game).

With this in mind, we can the ‘dismiss’ the sceptic who rejects these moral grammatical claims on similar grounds to that of the Cartesian sceptic.

## A Comparison between Moral Scepticism and Solipsism

Russell famously remarks of a logician he knew who claimed to be a solipsist, arguing that her position was contradicted by her practices and implicit beliefs:

“[Solipsism is] psychologically impossible to believe, and is rejected in fact even by those who mean to accept it. I once received a letter from an eminent logician, Mrs. Christine Ladd-Franklin, saying that she was a solipsist, and was surprised that there were no others. Coming from a logician and a solipsist, her surprise surprised me. The fact that I cannot believe something does not mean that it is false, but it does mean that I am insincere and frivolous if I pretend to believe it. Cartesian doubt has a value as a means of articulating our knowledge and showing what depends on what , but if carried too far it becomes a mere technical game in which philosophy loses seriousness. Whatever anybody, even I myself, may argue to the contrary, I shall continue to believe that I am not the whole universe…” (Russell 1948, p.195)

I suggest that the ‘insincerity’ of the logician who both claims to be a solipsist and implicitly admits the existence of other minds is similarly reflected in the views and actions of our theoretical moral sceptic. What is interesting here, however, is that whilst there are very few self-proclaimed solipsists in academia, many philosophers (and other people) either do claim to be amoralists or moral sceptics of some brand, or to some extent accept the views of the moral sceptic (implicitly or otherwise). I will not here suggest a positive explanation as to this distinction, however I will argue that the difference is not to do with some substantial philosophical difference between the two positions. That is, I will argue that moral scepticism is just as ‘psychologically impossible’ as solipsism, and hence moral sceptics risk running into the same ‘insincerity’ of Russell’s friend.

We can see this if we examine just the extent to which moral language is embedded in our everyday thought processes. Consider the following:

- I shouldn’t smoke, (since I care about my health)
- I shouldn’t move my pawn, (since I want to win this chess game)
- I shouldn’t kill my patients, (since I am a doctor)
- I shouldn’t kill someone (because I recognise them as fellow human beings)

According to the outline I have previously suggested, the same instrumental reasoning processes underline all of these examples, and they follow from various kinds of normative or moral practices, where such rules are recognised within a certain group or community. It seems that if we are to ‘sincerely’ reject these claims as meaningless, we would have to totally distance ourselves from any language-games with any normative content. This would involve not engaging in any social interactions where one could make normative claims like ‘you shouldn’t have done that’, ‘you should be careful’ or ‘I wouldn’t do that if I were you’. Moral sceptics who do hold all normative and moral claims to be false or nonsensical therefore seem to be taking as propositional or dubitable grammatical claims which are not to taken as such in practice.

To explain this point further, I will focus on Royce’s ‘The Myth of Morality’. Royce advocates a moral error theory over any absolute account of moral obligation, on the grounds that we cannot reconcile relativistic reasons that motivate particular individuals to actions, and the moral reasons that ought to motivate us or some “ideal observer” (Royce 2001, p. 8). The result is “alienat[ion] [of] the agent from rationality” (Royce 2001, p. 29), such that an agent has no reason to act according to this rational ideal of morality. In short, there is no absolute moral framework which has overriding moral force over our normative reasoning.

Royce compares the nature of these moral frameworks with some out-dated or problematic frameworks which we reject, such as the concept of phlogiston and the Maori word ‘tapu’. Royce describes these as “mistaken frameworks” since no assertions of the form ‘ϕ is phlogiston’ or ‘ϕ is tapu’ can be true (Royce 2001, pp. 23–26). Here we can begin to see how the Wittgensteinian approach I offer differs from Royce’s. Whilst scientific notions like phlogiston seem to refer to some natural-kind or ontological thing in order to be true, useful or correct, the same does not necessarily apply to moral language-games. If we instead take certain tautological and practical grammatical remarks as given (as ordinary language users do), we do not need to search for any absolute or ontological/metaphysical states of affairs which make them true or universally normatively binding. For instance, let’s imagine a language game in which the statement ‘ϕ is tapu’ is an instance of practical grammar. This may be a “mistaken framework” for that community in the sense that the concept is impractical or inefficient, but this is just a question of pragmatics. We do not have to reject the concept as false or meaningless just because it does not latch on to anything metaphysical. For instance, it may be a valuable rule/ritual/heuristic in its own right. We do not need to ask ‘what is the meaning of tapu?’ any more than ‘what is the meaning of the number ‘five” (Wittgenstein 1953, §1).

If the broader structure of normative reasoning which I outlined in part 2 of this essay still remain in this possible language-game, then these instances of practical grammar can be recalibrated with this accordingly. This does not, however, beg the question of what tapu ‘really is’ from a metaphysical standpoint. All we need to assume here for the sake of moral inquiry is the way that the term is used. This is even clearer if we look to examples of tautological grammar. Statements like ‘I should do the right thing’ or ‘I should act in my communities interests’ do not need to be made in reference to any further framework at all, (pragmatic or otherwise), and are simply institutional rules. Whilst Royce, (and presumably many ordinary language users), do not interpret moral claims as institutional, if we take the Wittgensteinian route this is simply a misinterpretation of language, where grammatical reminders are mistaken for propositional claims. (Royce 2001, p. 33) (Wittgenstein 1953, §251).

It is perhaps surprising how much of this essay is actually consistent with Royce’s claims. For instance, we seem to largely agree about the relativistic nature of morality, as something which is limited by institutional facts and language games. The dispute largely seems to rest on a simple question of how we define morality – as institutional or necessarily absolute. The reason for this is that the approach I have given does not exactly put forward a philosophical argument against the plausibility of Royce’s (or other moral sceptic’s) arguments. I have instead provided reasons to shift the emphasis of moral philosophy away from foundational questions about the metaphysical nature of morality, and instead focus on moral language as and how it is used by people. Royce may provide a substantial critique of rationalist philosophical accounts of morality, but this does not really speak to the moral grammar and language-games that people actually engage in.

## Conclusion

Moral scepticism is a widely held stance in both academic, and in wider cultural contexts^13^.^14^ I have argued that this scepticism is misplaced in much the same way as the Cartesian sceptic, since moral scepticism scrutinises grammatical moral claim which are not to be taken as propositional facts about the world, but rather as rules or heuristics about how we use moral language. Moreover, it seems that most people do take such grammatical claims for granted in their practices, even if this is not reflected in their philosophical standpoints about the matter. If we want moral inquiry to speak to the practical concerns and issues in moral language-games, we therefore need to push the problems of the moral sceptic aside by simply saying that some normative frameworks do exist where others don’t. That is, certain individuals or groups just *do* care about certain things, and hence we can make conditional claims about what these people ought to do, given their interests. There is no need for a universal, objective framework of morality in order for moral claims to be useful and have a truth value.

## Footnotes

1 Note that the moral scepticism I want to discuss in this paper is distinct from some accounts of *relativism* where moral statements can be made according to specific contextual frameworks (e.g. (Harman 2012)) – the moral sceptic goes further than this and says that any contextual framework is as good as any other, and hence any moral statement is just as ‘true’ as any others. Hence, no one is in any position to make any moral claims/judgements, even with cultural or circumstantial qualifications.

2 This is not to say that Wittgenstein’s account of language acquisition has no value as an empirical hypothesis, only that for the purposes of his epistemology it needn’t be taken as such.

3 This is because some recognition/understanding of what it means to not be in pain is needed to recognise what it means to be in pain.

4 One might at this point ask why a person consisting of multiple temporal parts can’t constitute their own public language. This might, I would suggest, be possible after the individual’s capacity for language has already been developed. Before this, however, the individual has still not been exposed to any existing language games and so the ability to recognise these temporal parts as existing in a wider social sphere does not exist.

5 Note, that for this reason, this distinction is not subject to Quine’s critique of the analytic/synthetic distinction, since we are not denying that both types of grammar can be reduced to ‘empirical’ practices, or trying to make any metaphysical distinction. (Quine 1951).

6 By instrumental reasoning I mean a form of reasoning according to which we ought to take the most useful/efficient means of achieving a particular desired end. See (Korsgaard 2008).

7 Of course, the question of what these values should be in the first place is another question. I will aim to address this question to some extent in a later essay.

8 For instance, I might think that I value x when I really value something else. What this would actually mean psychologically is unclear, but for now we might imagine cases where I might make a decision that I think should be in my best interests, but I later regret. The point is that ‘what do I *really* value?’ or ‘what does it mean for something to be in my interests’ are open questions.

9 As I understand it, Searle’s paper focuses on ‘descriptive’ statements which describe states of affairs, and ‘evaluative’ claims which evaluate these states of affairs according to some human/societal values. My paper, however, focuses on normative claims which attempt to say something about the way things *should* be. For instance, ‘my car is a good car’ is used by Searle as an example of an evaluative claim (Searle 1964, p. 53) but it is not a normative one by my definition.

10 For a defence of how grammatical claims like these are ‘normative’, see (Hacker 2012).

11 This, I believe, does not contradict Searle’s conclusion, but is simply referring to a slightly different problem than the one discussed in his paper.

12 I say ‘most’ rather than ‘all’ because, even though I can’t think of any actual counterexamples, there is no reason in theory that means no possible human language-games could deny these grammatical claims.

13 For example, MacIntyre argues that “The unrecognized philosophical power of emotivism is one clue to its cultural power” (MacIntyre 1981).

14 For some examples of this, see Midgley’s discussions on moral scepticism in P D James, Tom Stoppard, and Barbara Wootton (Midgley 1991).
